# IDH Mutations and Intraoperative 5-ALA Fluorescence in Gliomas: A Systematic Literature Review with Novel Exploratory Hypotheses on the Modulatory Effect of Vorasidenib

**DOI:** 10.3390/cancers17183075

**Published:** 2025-09-19

**Authors:** Magdalena Rybaczek, Marek Jadeszko, Aleksander Lebejko, Magdalena Sawicka, Zenon Mariak, Tomasz Łysoń, Halina Car, Przemysław Wielgat

**Affiliations:** 1 Department of Neurosurgery, Medical Univeristy of Bialystok, 15-276 Bialystok, Poland; mjadeszko63@gmail.com (M.J.); alebejko@gmail.com (A.L.); zenon.mariak@umb.edu.pl (Z.M.); tomasz.lyson@umb.edu.pl (T.Ł.); 2 Department of Analysis and Bioanalysis of Medicines, Faculty of Pharmacy with the Division of Laboratory Medicine, Medical University of Bialystok, 15-222 Bialystok, Poland; magdalena.sawicka@umb.edu.pl; 3 Department of Clinical Pharmacology, Medical Univeristy of Bialystok, 15-274 Bialystok, Poland; hcar@umb.edu.pl (H.C.); przemyslaw.wielgat@umb.edu.pl (P.W.)

**Keywords:** glioma, IDH, 5-ALA, fluorescence, vorasidenib

## Abstract

**Background**: Fluorescence-guided surgery using 5-aminolevulinic acid (5-ALA) enables the intraoperative visualization of glioma. However, its effectiveness varies based on tumor subtype and molecular profile, posing challenges for achieving complete resection. Our systematic review aims to explore the relationship between IDH mutation status and intraoperative fluorescence visualization. Importantly, this is the first study to propose that vorasidenib, an emerging IDH-targeting agent, could enhance 5-ALA-guided surgery, marking a novel direction for translational research. **Methods**: A systematic literature search was conducted using the PubMed, Cochrane Library, Scopus and Web of Science databases up to May 2025, following PRISMA guidelines. The primary outcomes included fluorescence detection rates across different glioma subtypes and their correlation with IDH mutation status. Secondary outcomes comprised surgical efficacy measures such as gross total resection (GTR), overall survival (OS), and progression-free survival (PFS). Additionally, we analyzed the metabolic consequences of IDH mutations and evaluated the potential role of vorasidenib in enhancing 5-ALA-induced fluorescence. **Results**: Seven studies including 621 patients included in the final analysis. Fluorescence detection was nearly universal in WHO grade 4 gliomas (94–100%), but lower in grade 3 (43–85%) and rare in grade 2 (7–26%). Several cohorts reported reduced fluorescence in IDH-mutant gliomas, although this was not consistent across all studies. In high-grade gliomas, visible fluorescence correlated with higher GTR rates and, in some series, longer OS. Conversely, in lower-grade IDH-mutant gliomas, fluorescence did not increase GTR and was associated with worse PFS and OS. **Conclusions**: The effectiveness of 5-ALA-guided fluorescence in glioma surgery is significantly influenced by both tumor grade and IDH mutation status. Vorasidenib may represent a potential avenue for modulating tumor metabolism and enhancing intraoperative fluorescence in IDH-mutant gliomas, a hypothesis that warrants further experimental validation.

## Simple Summary

Gliomas are primary brain tumors in which the extent of surgical resection is a critical factor influencing patient prognosis. Fluorescence-guided surgery with oral administration of 5-aminolevulinic acid enables the intraoperative visualization of tumor tissue, thereby facilitating more complete resections. Nevertheless, the intensity of fluorescence differs substantially between tumor grades and molecular subtypes. In particular, gliomas harboring mutations in the isocitrate dehydrogenase gene frequently exhibit weak or absent fluorescence, which complicates the intraoperative identification of tumor margins and may limit surgical efficacy. Our systematic review presents the correlation between fluorescence intensity, tumor grade, and molecular status, and emphasizes its impact on resection rates and survival outcomes. Furthermore, we introduce an exploratory hypothesis that vorasidenib, a novel inhibitor of mutant isocitrate dehydrogenase recently approved for clinical use, could restore metabolic balance and enhance fluorescence in these tumors. This concept may open new avenues for integrating targeted metabolic therapy with fluorescence-guided neurosurgery.

## 1. Introduction

Gliomas constitute a heterogeneous group of central nervous system tumors characterized by distinct clinical courses, molecular profiles, and prognoses. According to the latest CNS World Health Organization (WHO) classification, a pivotal diagnostic criterion is the presence of mutations in the isocitrate dehydrogenase (IDH) genes, IDH1 or IDH2 [1]. Gliomas with IDH mutations (IDH-mutant) are generally associated with more favorable clinical outcomes and prolonged survival compared to IDH-wildtype gliomas, which tend to be more aggressive and confer a worse prognosis [2].

Neurosurgical resection remains the cornerstone of initial glioma management, with the extent of tumor removal being a critical determinant of both progression-free survival (PFS) and overall survival (OS) [3].

This association has been consistently confirmed in large-scale analyses and meta-analyses, demonstrating that greater extent of resection correlates with improved overall survival in glioblastoma [4,5]. However, the infiltrative nature of gliomas often complicates the intraoperative delineation of tumor margins, limiting the feasibility of achieving gross total resection (GTR). One of the most impactful advances in glioma surgery has been the introduction of 5-aminolevulinic acid (5-ALA)—mediated fluorescence-guided resection [6,7].

Following oral administration and subsequent cellular uptake, 5-ALA is converted to porphobilinogen (PBG), which is further metabolized via the heme biosynthesis pathway through a cascade of enzymatic reactions catalyzed by coproporphyrinogen oxidase (CPOX) and protoporphyrinogen oxidase (PPOX), ultimately yielding protoporphyrin IX (PpIX), a compound with fluorescent properties. Within mitochondria, PpIX serves as a direct precursor to heme and undergoes conversion via the insertion of ferrous iron by ferrochelatase (FECH) [8]. In many high-grade gliomas, dysregulated porphyrin metabolism and reduced FECH activity result in selective PpIX accumulation. This phenomenon is utilized intraoperatively, allowing for accurate identification and targeted resection of malignant tissue. A randomized controlled trial has shown that 5-ALA-guided fluorescence significantly increases GTR rates and improves outcomes, particularly in high-grade gliomas [9].

Nevertheless, the clinical usefulness of 5-ALA varies considerably between glioma subtypes. High-grade tumors, especially glioblastomas, usually display strong and consistent fluorescence, whereas low-grade gliomas are widely regarded as non-fluorescent after 5-ALA administration. This limitation is particularly risky in IDH-mutant gliomas, which, despite their generally favorable prognosis, exhibit distinct metabolic alterations that impair PpIX accumulation. The mutation in the isocitrate dehydrogenase gene promotes a neomorphic enzymatic reaction leading to the production of the oncometabolite 2-hydroxyglutarate [10]. This metabolite interferes with mitochondrial function and inhibits key enzymes of the heme biosynthesis pathway, such as CPOX and PPOX [11,12]. As a result, intraoperative fluorescence is markedly reduced or absent, making tumor tissue difficult to distinguish from normal brain parenchyma. Thus, an increased risk of incomplete resection may substantially diminish the prognostic advantage typically associated with this glioma subtype.

In recent years, a novel and evolving oncological strategy has focused on targeting IDH mutations as a therapeutic approach in glioma treatment. Vorasidenib—a selective oral inhibitor of mutant IDH1 and IDH2 enzymes—has emerged as a novel therapeutic option. It is the first IDH-directed glioma therapy approved by the U.S. Food and Drug Administration (FDA) [11]. Clinical trials, including the phase 3 INDIGO (INdividualized Treatment for IDH1-mutant Glioma With Vorasidenib) study, have demonstrated that Vorasidenib significantly prolongs PFS in patients with WHO grade 2 IDH-mutant gliomas, delaying the need for more invasive therapeutic interventions [12].

The therapeutic effect of vorasidenib is mediated through the selective inhibition of mutant IDH1 and IDH2 enzymes, thereby lowering intracellular concentrations of 2-HG, which is known to disrupt the heme biosynthesis pathway [13]. These observations suggest that vorasidenib, through the restoration of mitochondrial metabolism and suppression of 2-HG accumulation, may enhance PpIX production and improve intraoperative visualization in IDH-mutant gliomas, which are relatively resistant to 5-ALA-induced fluorescence. However, it must be emphasized that no preclinical or clinical studies have directly evaluated the relationship between vorasidenib and 5-ALA fluorescence, and the proposed mechanism remains hypothetical. While this interaction requires empirical validation, it may represent a promising avenue for enhancing the efficacy of 5-ALA-guided surgical resection and ultimately improving PFS and OS in patients with IDH-mutant gliomas.

In this context, we conducted a systematic review to synthesize current evidence on intraoperative 5-ALA-induced fluorescence across glioma grades and molecular subgroups, with particular emphasis on the role of IDH mutation status. Based on this foundation, we propose an exploratory hypothesis that vorasidenib, through its metabolic effects, may enhance PpIX accumulation and thereby improve intraoperative fluorescence in IDH-mutant gliomas. We present, for the first time, this hypothesis as a speculative framework intended to provide a rationale for future translational studies aimed at bridging targeted metabolic therapy with fluorescence-guided resection.

## 2. Materials and Methods

### 2.1. Search Strategy

The presented systematic review was performed according to the Preferred Reporting Items for Systematic Reviews and Meta-Analysis (PRISMA) guidelines. This systematic review has not been registered in any database.

**Figure 1:**
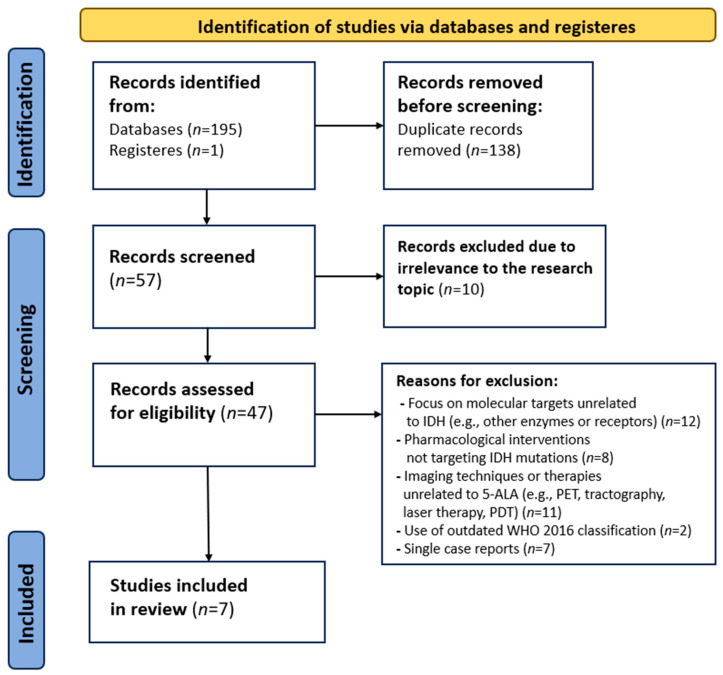
PRISMA flow diagram illustrating the study selection process for the systematic literature review.

The PubMed, Cochrane Library, SCOPUS and Web of Science databases were searched using the terms combinations: “5-ALA + IDH”, “5-aminolevulinic + IDH”, “vorasidenib + 5-ALA”, “vorasidenib + 5-aminolevulinic” up to May 2025. A schematic PRISMA flow diagram depicting the selection of the studies included in this review is presented in Figure 1.

The choice of search terms was intentionally limited to ensure clinical relevance to the review question. Pilot searches with broader terms (e.g., “protoporphyrin IX”, “fluorescence-guided resection”) retrieved predominantly basic science papers or highly heterogeneous surgical reports not specific to intraoperative 5-ALA fluorescence. Importantly, because we did not restrict keywords to title/abstract fields, studies using broader descriptors but also mentioning 5-ALA were captured. This approach minimized irrelevant results while maintaining sensitivity for clinically meaningful studies.

### 2.2. Study Selection and Data Extraction

Two authors independently screened the literature by reviewing the full texts of eligible manuscripts to extract data on authorship and publication year, study methodology, sample size, glioma subtypes classified by WHO grade, the percentage of 5-ALA-induced fluorescence observed across glioma grades, associations between fluorescence and IDH mutation status, the clinical significance of fluorescence in the context of surgical resection, and technical details such as the type of operative microscope and fluorescence detection techniques used. Study limitations and available long-term outcomes, including survival data and treatment efficacy, were also analyzed.

### 2.3. Inclusion Criteria

The inclusion criteria were defined according to the PICOS framework:

**P (Population):** Patients diagnosed with WHO grade 2–4 gliomas, including both primary and recurrent cases, who underwent surgical resection.

**I (Intervention):** Use of 5-ALA-induced fluorescence during neurosurgical tumor resection.

**C (Comparison):** Comparison between fluorescence-guided surgery and standard white-light surgery, with particular emphasis on IDH mutation status and the extent of resection achieved with and without fluorescence guidance.

**O (Outcomes):** Primary outcomes included fluorescence detection rates across glioma subtypes and their correlation with IDH mutation status. Secondary outcomes encompass surgical efficacy measures—such as gross total resection (GTR)—and long-term oncological outcomes, including overall survival (OS) and progression-free survival (PFS).

**S (Study Design):** Eligible studies included original research articles and comparative studies (e.g., case-control, cohort studies, and randomized clinical trials) published in English up to May 2025.

### 2.4. Exclusion Criteria

As part of the systematic literature review, studies were excluded if they focused on variable epigenetic profiles or mutations in enzymes or receptors other than IDH, investigated the effects of unrelated pharmacological agents, assessed outcomes based on PET imaging, tractography, laser therapy, or photodynamic therapy; relied on the previous WHO classification (2016, 4th edition) or were limited to case reports.

### 2.5. Bias Considerations

Independent dual screening with consensus was used to minimize reviewer bias. Only studies applying the WHO 2021 CNS classification (not the 2016 edition) were included. Remaining risks include selection/database-coverage bias, language bias (English only), and measurement bias from non-standardized fluorescence assessment.

### 2.6. Statistical Analysis

Due to the substantial heterogeneity of study methodologies (non-standardized fluorescence grading, variable IDH testing, and inconsistently reported outcomes), a formal meta-analysis was not feasible. In accordance with PRISMA recommendations, we therefore adopted a descriptive synthesis of the extracted data. Results from the included studies were summarized using basic summary statistics, including frequencies, percentages, and ranges. Statistical outcomes, such as p-values and confidence intervals, are reported as presented in the original publications. No additional statistical analyses were conducted due to heterogeneity in study designs and reported outcomes.

## 3. Results

### 3.1. Study Selection

A literature search conducted by PRISMA methodology yielded a total of 195 records: 99 related to “5-ALA + IDH” and 96 to “5-aminolevulinic acid + IDH.” No results were identified for either “vorasidenib + 5-ALA” or “vorasidenib + 5-aminolevulinic acid.”

After the removal of 138 duplicate records, titles and abstracts of the remaining studies were screened, resulting in the exclusion of 10 records due to irrelevant subject matter. Full-text review was then conducted for the remaining 47 articles, of which 40 were excluded based on criteria outlined in the Exclusion Criteria section. Ultimately, seven articles were included in the final analysis [14,15,16,17,18,19,20]. A PRISMA flow diagram illustrating the selection process is shown in Figure 1.

The studies, published between 2015 and 2025, were conducted in Italy (*n* = 3), Germany (*n* = 1), Japan (*n* = 1), Korea (*n* = 1), and Brazil (*n* = 1). All of them evaluated the use of 5-ALA–induced fluorescence in glioma surgery, including both primary and recurrent tumors across WHO grades 2 to 4. The final selection consisted of seven retrospective cohort studies.

### 3.2. Intraoperative Fluorescence and Its Association with Tumor Grade and IDH Mutation

In the seven studies included in this review, a total of 621 patients were analyzed, encompassing both primary and recurrent gliomas across WHO grades 2 to 4. The individual cohorts were as follows: 112 patients with lower-grade IDH-mutant gliomas (69 astrocytomas and 43 1p/19q-codeleted oligodendrogliomas, WHO grades 2–3) [14], 44 patients with newly diagnosed glioblastoma (WHO grade 4), including 16 with IDH mutations and 28 without IDH mutations [15], 179 patients with WHO grade 2 or 3 gliomas (113 WHO grade 2, 66 WHO grade 3) [16], 25 patients with newly diagnosed glioblastoma without IDH mutations [17], 58 patients with recurrent gliomas, divided into a 5-ALA group (*n* = 58) and a control group (*n* = 65) [18], 104 patients, including 68 who underwent initial surgery and 36 who were reoperated for gliomas WHO grades 2–4 [19], and 99 patients with newly diagnosed glioblastoma without IDH mutations (WHO grade 4) [20].

Fluorescence detection rates were reported across glioma grades. In WHO grade 4 gliomas, fluorescence was observed in 94% to 100% of cases [15,17,18,19] and in 100% in another large cohort [20]. In grade 3 gliomas, detection rates ranged from 43% to 85% [14,16,18,19], while in grade 2 gliomas, rates were substantially lower, typically between 12% and 26%, with some series reporting values around 25% [14,16,19].

Most investigations provided subgroup analyses stratified by IDH mutation status, generally demonstrating reduced fluorescence in IDH-mutant gliomas compared to tumors without IDH mutations. However, some series did not confirm a statistically significant correlation [14]. Surgical procedures included both primary resections and re-operations for recurrent disease. Standard neurosurgical microscopes equipped with fluorescence modules, such as the Carl Zeiss BLUE 400 system or Leica M530 OHX with FL400/FL560 filters, were used in the included studies. Characteristics of the analyzed cohorts are summarized in Table 1.

### 3.3. Surgical and Oncological Outcomes

The potential of fluorescence in improving patient outcomes is significant. Gross total resection was significantly more achievable in tumors exhibiting strong fluorescence. In the study by Müther et al. [16], GTR was achieved in 96.8% of patients with visible fluorescence, compared to 80.5% in non-fluorescent cases. Similarly, Specchia et al. [15] reported increased GTR rates in the group without IDH mutations (which had higher fluorescence intensity), with a statistically significant difference (*p* < 0.05). In another large series of glioblastoma patients without IDH mutations, a ≥90% extent of resection was achieved in 87.5% of cases operated with 5-ALA, 77.3% with fluorescein sodium, and 80% with the combined use of both fluorophores, with no significant differences between groups (*p* = 0.783) [20].

In terms of progression-free survival (PFS) and overall survival (OS), Kim et al. [17] observed that patients with resection of all fluorescent tissue had significantly longer OS (median 28.8 months) compared to those with residual fluorescent tissue (median 19.1 months, *p* = 0.026). Other studies provided a more nuanced view: in a series of lower-grade IDH-mutant gliomas [14], fluorescence positivity did not correlate with higher GTR rates (overall GTR 71%) but was instead associated with shorter OS (~2.5-fold higher risk of death, *p* = 0.009) and PFS (~2.5-fold higher risk of progression, *p* = 0.004). In glioblastoma, median OS was reported as 20.0 months for patients operated with 5-ALA, 12.3 months for those with fluorescein sodium, and 18.1 months for the combined fluorophores, with an overall median survival of 14.9 months; these differences did not reach statistical significance [20].

This paradoxical finding does not indicate that fluorescence itself worsens outcomes, but rather that intraoperative fluorescence in lower-grade gliomas tends to occur in tumors with higher biological aggressiveness [14]. In this context, 5-ALA and FS act as markers of poor prognosis rather than direct predictors of surgical efficacy.

In contrast, IDH-mutant gliomas without fluorescence, typically less aggressive in their behavior, were associated with slightly lower GTR rates but more favorable long-term outcomes. Nevertheless, across studies, complete resection of all fluorescent tissue consistently correlated with improved PFS and OS when present, underscoring the clinical value of reliable intraoperative visualization. Large surgical series further support that maximal safe resection, even beyond conventional contrast-enhancing boundaries, is associated with incremental survival benefit [21].

### 3.4. Comprehensive Findings from the Literature Review

Bianconi et al. [14], in a retrospective cohort of 112 patients with IDH-mutant lower-grade gliomas (astrocytomas and oligodendrogliomas, WHO grades 2–3), reported intraoperative 5-ALA-induced fluorescence in 25% of grade 2 and 43% of grade 3 tumors. Fluorescein sodium demonstrated even lower detection rates, occurring in 12% of grade 2 and 23% of grade 3 cases. These observations underscore the marked contrast between lower-grade gliomas and glioblastoma, where intraoperative fluorescence is almost universally observed. Detection rates in lower-grade gliomas remain modest and appear to increase in parallel with tumor grade.

The occurrence of visible fluorescence in this subgroup should be regarded not primarily as a technical adjunct facilitating resection, but rather as a potential surrogate marker of underlying biological aggressiveness. Focal areas of intraoperative fluorescence within otherwise low-grade tumors may represent regions of increased anaplasia or early malignant transformation. Identifying these areas enables targeted resection and histopathological sampling, thereby improving diagnostic accuracy and supporting therapeutic decisions in the postoperative setting.

In a retrospective single-center study, Specchia et al. [15] examined the correlation between intraoperative 5-ALA fluorescence and molecular markers in patients with histologically confirmed glioblastoma (WHO grade 4). The study included 44 patients who underwent fluorescence-guided resection using a Zeiss Pentero microscope equipped with a 400 nm ultraviolet filter. Fluorescence intensity was graded using a standardized three-point scale (Grade 0–2) and independently assessed by three blinded observers, resulting in near-perfect inter-rater agreement (Fleiss’ Kappa = 0.92).

The results showed that fluorescence intensity varied according to IDH mutation status. In the IDH-wildtype group (*n* = 28), 46.4% of tumors demonstrated strong fluorescence (Grade 2), 25% showed weak fluorescence (Grade 1), and 28.6% exhibited no fluorescence (Grade 0). In contrast, within the IDH-mutant group (*n* = 16), only 6.25% demonstrated strong fluorescence, while 50% showed no fluorescence at all. Although this difference did not reach statistical significance (*p* = 0.25), the authors observed a trend suggesting that IDH-wildtype tumors exhibit increased 5-ALA uptake and metabolic activation, consistent with their higher proliferative activity.

Additionally, no statistically significant associations were found between fluorescence grade and other biological or clinical factors, including MGMT (O^6^-Methylguanine-DNA Methyltransferase) promoter methylation status, patient age, sex, or mitotic index (*p* = 0.93). The study supports the role of 5-ALA as a metabolic tracer, particularly in IDH-wildtype glioblastomas, where fluorescence may assist in delineating tumor margins and optimizing resection strategies. However, the authors emphasize that 5-ALA fluorescence cannot replace molecular diagnostics, and its limited utility in IDH-mutant gliomas should be taken into consideration during surgical planning.

It is important to note that the study did not evaluate outcome measures such as GTR, OS, or PFS. Limitations include the small sample size, lack of objective quantitative assessment of fluorescence (e.g., PpIX spectroscopy), and potential selection bias. While 5-ALA remains a valuable intraoperative adjunct, its variable reliability—particularly in IDH-mutant tumors—highlights the importance of multimodal guidance and personalized surgical decision-making.

The study by Müther et al. [16] retrospectively evaluated 179 patients with newly diagnosed WHO grade 2 and 3 diffuse gliomas to identify clinical and radiological predictors of 5-ALA-induced intraoperative fluorescence. Using a logistic regression model validated via leave-one-out cross-validation (LOOCV), the authors identified contrast enhancement (CE) patterns on preoperative MRI-graded from CE 0 (no enhancement) to CE 3 (abundant enhancement)—as the only significant predictor of visible fluorescence.

The presence and intensity of contrast enhancement predicted intraoperative fluorescence with an overall accuracy of 91.9%, with subgroup accuracies of 81.0% for WHO grade 2 gliomas and 93.0% for WHO grade 3 gliomas. Visible fluorescence was observed in 75–85% of grade 3 tumors and 16–20% of grade 2 tumors, aligning with previously reported ranges. The probability of intraoperative fluorescence increased proportionally with the degree of contrast enhancement. For instance, non-enhancing tumors (CE 0) had a 91.2% probability of lacking fluorescence, while tumors with abundant enhancement (CE 3) had a 91.3% probability of demonstrating fluorescence.

Additionally, fluorescence showed a significant correlation with the MIB-1 proliferation index (*p* < 0.001), but no association was found with IDH mutation status (*p* = 0.814), 1p/19q codeletion, or MGMT promoter methylation. All patients received 5-ALA (20 mg/kg) orally 3–4 h before surgery and underwent fluorescence-guided resection using Zeiss Pentero or Pentero 900 microscopes equipped with the BLUE400 module. Intraoperative fluorescence assessment was standardized in terms of microscope settings, working distance, and ambient lighting conditions.

Although the study did not include outcome measures such as gross total resection GTR, OS, or (PFS, it underscores the predictive value of MRI contrast enhancement for intraoperative fluorescence. This finding highlights the potential utility of 5-ALA in identifying biologically aggressive tumor regions in intermediate-grade gliomas.

In their institutional series, Kim et al. [17] investigated 25 patients with newly diagnosed IDH-wildtype glioblastoma who underwent GTR of contrast-enhancing lesions and strongly fluorescent regions following 5-ALA administration. A total of 136 biopsies from the resection cavity and 29 from the ventricular wall were histologically analyzed and correlated with intraoperative fluorescence intensity. Residual fluorescence was intraoperatively assessed using the Zeiss BLUE400 filter system and categorized as either pink (vague fluorescence) or blue (no visible fluorescence).

Among pink-fluorescent areas, 50% demonstrated definitive infiltration by tumor cells, while only 29% of blue-fluorescent samples showed such infiltration. However, there was no statistically significant association between residual fluorescence and histopathological tumor cell presence in either the resection cavity (*p* = 0.199) or ventricular wall (*p* = 0.704).

The median PFS was 12.5 ± 2.1 months, and the median OS was 21.1 ± 3.5 months. Univariate analysis revealed that the presence of infiltrating tumor cells in peritumoral regions was significantly associated with shorter PFS (*p* = 0.002) and OS (*p* = 0.027). In multivariate Cox regression, the absence of definitive tumor infiltration remained an independent predictor of improved survival outcomes (PFS: HR 0.184, *p* = 0.012; OS: HR 0.124, *p* = 0.050). In contrast, the presence or absence of residual fluorescence alone did not correlate significantly with either PFS or OS.

These findings suggest that while residual 5-ALA fluorescence may offer intraoperative guidance, it does not reliably predict microscopic tumor infiltration. Instead, histological confirmation of infiltrating tumor cells beyond the enhancing core appears to carry stronger prognostic significance. This supports the role of supramarginal resection in selected patients, even in the absence of visible fluorescence.

In a retrospective analysis, Hickmann et al. [18] evaluated 63 fluorescence-guided surgical procedures performed on 58 patients with recurrent WHO grade 3 or 4 gliomas to assess the feasibility and prognostic impact of 5-ALA-guided resection in the recurrent disease setting. Fluorescence was observed intraoperatively in 84.1% of all surgical procedures, with higher detection rates in WHO grade 4 tumors (94.7%) compared to WHO grade 3 tumors (68.0%).

Non-fluorescent tumors were significantly more likely to be of WHO grade 3, IDH-mutant status, and exhibit oligodendroglial histology. Molecular profiling demonstrated a strong association between fluorescence and IDH mutation status: 100% of non-fluorescent tumors were IDH-mutant, whereas only 48.9% of fluorescent tumors harbored IDH mutations (*p* = 0.005). A complete resection—defined as ≥98% extent of resection (EOR)—was achieved in 58.8% of fluorescent tumors, compared to 50.0% in non-fluorescent cases, although this difference did not reach statistical significance. Notably, patients in whom all fluorescent tissue was removed demonstrated a significantly higher mean EOR (96.8% vs. 85.7%, *p* = 0.014) and experienced improved overall survival (*p* = 0.038). While 5-ALA-guided surgery did not significantly impact PFS, it was associated with a longer median OS compared to the control group (17.6 months vs. 14.6 months, *p* = 0.025). All procedures were performed using a BLUE400 fluorescence microscope with Brainlab navigation. For tumors located in eloquent brain regions, functional neuronavigation and intraoperative neurophysiological monitoring were utilized. These findings highlight the utility of 5-ALA fluorescence for enhancing intraoperative visualization in the setting of recurrent gliomas and suggest a prognostic benefit when complete resection of fluorescent tissue is achieved.

Another study, by Ohba et al. [19] examined intraoperative 5-ALA fluorescence in a cohort of 104 patients undergoing surgery for WHO grade 2–4 gliomas. Their retrospective study focused on the correlation between fluorescence intensity and IDH mutation status. Fluorescence was observed in 78.8% (82 out of 104) of cases. When stratified by WHO grade, there was a notable increase in fluorescence positivity with higher tumor grades: 26.3% (5 out of 19) for WHO grade 2, 76.9% (20 out of 26) for grade 3, and 96.6% (57 out of 59) for grade 4 tumors.

Analysis of molecular markers indicated that IDH1 and IDH2 mutations, which were present in 35.6% of tumors, were significantly associated with reduced intraoperative fluorescence (51.4% in IDH-mutant tumors compared to 94.0% in IDH-wildtype tumors, *p* < 0.01). Among lower-grade gliomas (WHO grades 2–3), IDH-mutant tumors showed a trend toward decreased fluorescence (48.6% vs. 80.0%), although this did not reach statistical significance (*p* = 0.08). Additionally, no significant associations were found between fluorescence status and patient age, sex, tumor location, or MGMT promoter methylation.

In vitro analyses using glioma cell lines engineered to express IDH1 mutations demonstrated elevated levels of ferrochelatase (FECH) and heme oxygenase-1 (HO-1), enzymes involved in heme metabolism. This expression pattern suggests increased degradation PpIX in IDH-mutant cells. The molecular phenotype correlated with progressively diminished fluorescence across serial cell passages, further indicating that IDH mutation status may impair PpIX accumulation and thereby reduce intraoperative fluorescence in these tumors.

All patients underwent surgery using a Zeiss OPMI Pentero microscope with the BLUE400 filter. The study did not assess the extent of resection or long-term clinical outcomes, including PFS and OS.

Finally, in a retrospective single-center study, Zeppa et al. [20] evaluated the effectiveness of different fluorophores in fluorescence-guided resection of histologically confirmed glioblastoma (WHO grade 4) without IDH mutations. The study included 99 consecutive patients who underwent surgery using 5-ALA, sodium fluorescein, or a combination of both agents. Patients were stratified into three groups: 40 received 5-ALA, 44 received fluorescein sodium, and 15 were operated on with both fluorophores.

The analysis demonstrated that intraoperative fluorescence was consistently detectable across all groups, reflecting the high prevalence of fluorescence in glioblastoma. Extent of resection ≥ 90% was achieved in 87.5% of cases with 5-ALA, 77.3% with fluorescein sodium, and 80% with the combined approach, with no statistically significant differences between groups (*p* = 0.783). Functional outcomes were comparable, with similar rates of postoperative Karnofsky Performance Status decline across groups (*p* = 0.270).

Overall survival for the entire cohort was 14.9 months. Median OS was 20.0 months in the 5-ALA group, 12.3 months in the fluorescein sodium group, and 18.1 months in the combined group, with no significant survival differences observed (trend toward longer OS in the 5-ALA and combined groups; *p* = 0.071 in MGMT-stratified subgroups). The study therefore suggests that both fluorophores are effective and safe intraoperative tools for guiding glioblastoma resection, with comparable resection rates and functional outcomes. At the same time, potential survival advantages require further investigation.

Limitations include the retrospective design, lack of randomization, single-center setting, and relatively small subgroup sizes, particularly in the combined fluorophore cohort. Despite these constraints, the study contributes valuable evidence regarding the relative performance of 5-ALA and fluorescein sodium in fluorescence-guided surgery for glioblastoma. It supports their role as reliable adjuncts in optimizing the extent of resection without increasing postoperative morbidity.

## 4. Discussion

This literature review provides evidence that the intraoperative detection of 5-ALA-induced fluorescence is strongly influenced by WHO tumor grade, with fluorescence being almost universally observed in grade 4 gliomas [17,18,19,20] and substantially less frequent in lower-grade tumors [18,19]. In lower-grade gliomas, several studies reported notably reduced fluorescence in tumors with IDH mutations [18,19], whereas others did not confirm this association [14,15].

In glioblastoma without IDH mutations, intraoperative fluorescence was consistently detectable and enabled high rates of ≥90% resection with comparable functional outcomes [20].

These observations are consistent with a recent systematic review that specifically examined intraoperative fluorescence in lower-grade gliomas, synthesizing evidence from 16 clinical series employing 5-ALA as well as reports on alternative fluorophores including sodium fluorescein, indocyanine green, and tozuleristide [22]. That analysis demonstrated that visible 5-ALA fluorescence was present in only a minority of WHO grade II gliomas, with detection rates typically ranging between 7% and 15%. Notably, fluorescence in these tumors was not homogeneously distributed but tended to occur within intratumoral regions characterized by increased cellularity, enhanced proliferative activity, and angiogenic features. These findings suggest that, in the context of lower-grade gliomas, intraoperative fluorescence should be regarded less as a universal tool for surgical guidance and more as a surrogate marker of focal malignant progression.

Collectively, the available evidence indicates that 5-ALA-induced fluorescence is a reliable adjunct in the surgical management of high-grade gliomas, where it facilitates maximal safe resection. In contrast, its value in lower-grade gliomas appears limited. In this setting, the presence of intraoperative fluorescence may provide important prognostic information, reflecting areas of histological progression. However, it cannot be considered a consistent or comprehensive tool for guiding the extent of resection.

Our systematic review confirms that intraoperative 5-ALA fluorescence is strongly associated with WHO tumor grade and variably influenced by IDH mutation status. Based on this finding, we propose an exploratory hypothesis that the IDH inhibitor vorasidenib may modulate porphyrin metabolism and enhance intraoperative fluorescence in IDH-mutant gliomas. Importantly, this concept is speculative and has not yet been investigated experimentally.

**Figure 2:**
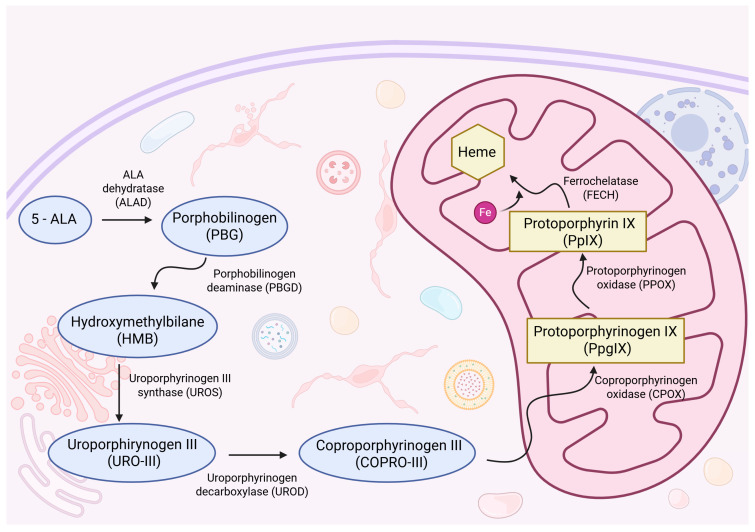
Schematic overview of the heme biosynthesis pathway, illustrating the enzymatic conversion of 5-ALA to protoporphyrin IX (PpIX) via cytosolic and mitochondrial intermediates. Created with BioRender.com. https://BioRender.com/dv7t31k (accessed on 6 August 2025).

As outlined in the introduction and presented in Figure 2, 5-ALA is metabolized through the mitochondrial heme biosynthesis pathway, ultimately leading to the accumulation of the fluorescent intermediate PpIX. The observed disparity in fluorescence between IDH-wildtype and IDH-mutant gliomas has raised important questions about the underlying metabolic alterations driven by IDH mutations.

**Figure 3:**
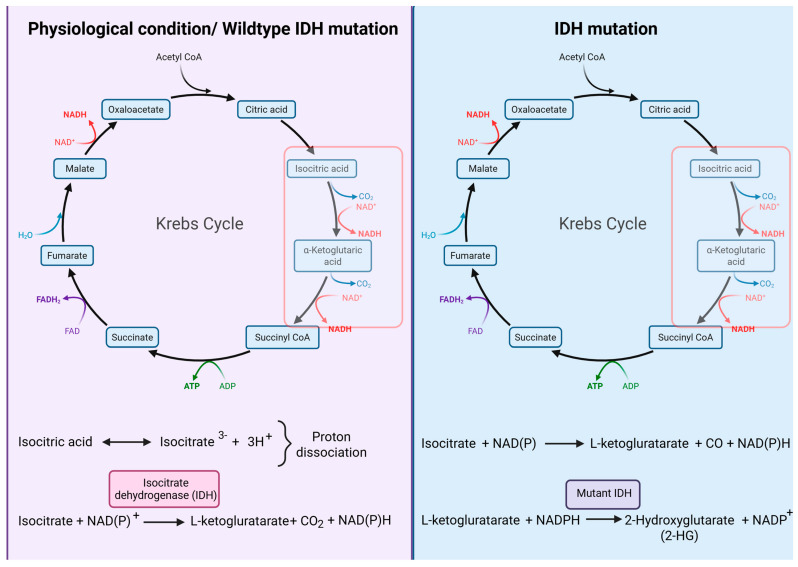
Comparison of physiological TCA cycle activity under wildtype IDH conditions with the reprogrammed metabolic flux in IDH-mutant cells, highlighting the neomorphic conversion of α-ketoglutarate to 2-hydroxyglutarate (2-HG) catalyzed by mutant IDH enzymes. Created with BioRender.com. https://BioRender.com/swpyo6h (accessed on 6 August 2025).

The reduced fluorescence observed in IDH-mutant gliomas has been associated with the intracellular accumulation of the oncometabolite 2-HG, which is produced as a result of the neomorphic enzymatic activity of mutant IDH1 and IDH2 isoforms [10]. This metabolic reprogramming alters the physiological flux of the tricarboxylic acid (TCA) cycle, thereby impairing mitochondrial respiration and redox homeostasis, as illustrated in Figure 3.

**Figure 4:**
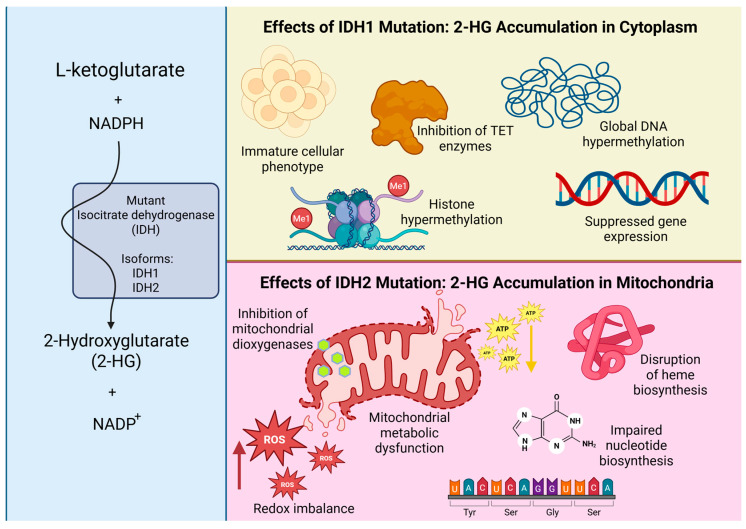
Compartment-specific effects of mutant IDH1- and IDH2-driven 2-hydroxyglutarate (2-HG) accumulation, illustrating distinct cytoplasmic and mitochondrial molecular disruptions, including epigenetic reprogramming, redox imbalance, and impaired biosynthetic processes. Created with BioRender.com. https://BioRender.com/72aqrdl (accessed on 6 August 2025).

IDH mutations profoundly affect tumor biology through epigenetic and metabolic mechanisms. While wild-type IDH enzymes support physiological cellular metabolism, mutant isoforms disrupt TCA cycle flux and promote the accumulation of 2-HG in distinct subcellular compartments, depending on the specific mutation. In IDH1-mutant tumors, 2-HG accumulates primarily in the cytoplasm, where it inhibits α-ketoglutarate-dependent dioxygenases, including DNA and histone demethylases, leading to widespread epigenetic dysregulation and global hypermethylation [23,24]. These alterations have been shown to suppress transcriptional programs governing cellular differentiation [24] and to promote invasive phenotypes, which can be partially reversed by pharmacological IDH inhibition [25]. By contrast, IDH2 mutations result in mitochondrial accumulation of 2-HG, which inhibits mitochondrial dioxygenases, increases reactive oxygen species (ROS) production, and disrupts mitochondrial metabolism. These effects impair biosynthetic pathways such as heme and nucleotide synthesis, which are essential for cellular proliferation [23,26]. Figure 4 illustrates the compartment-specific molecular consequences of 2-HG accumulation in IDH1- and IDH2-mutant gliomas.

This dual mechanism of epigenetic and metabolic disruption promotes an immature and less differentiated cellular phenotype characterized by impaired lineage commitment, reduced cellular proliferation rates, and altered tumor microenvironment interactions. These features are associated with slower tumor growth and may partly account for the more favorable prognosis typically observed in patients with IDH-mutant gliomas [2,23,26].

**Figure 5:**
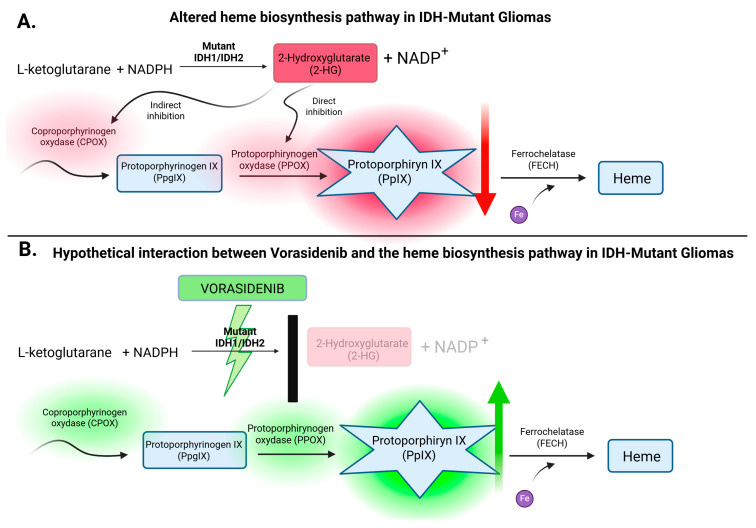
(**A**) Schematic representation of the disrupted heme biosynthesis pathway in IDH-mutant gliomas, illustrating the inhibitory effect of 2-hydroxyglutarate (2-HG) on mitochondrial enzymes CPOX and PPOX, resulting in reduced protoporphyrin IX (PpIX) accumulation. (**B**) Hypothetical model of vorasidenib-mediated restoration of 5-ALA–induced fluorescence through inhibition of mutant IDH activity and subsequent normalization of mitochondrial heme biosynthesis, including reactivation of CPOX and PPOX enzymes and enhanced PpIX production. Created with BioRender.com. https://BioRender.com/hom3680 (accessed on 6 August 2025).

Among the specific metabolic pathways affected by 2-HG accumulation, the mitochondrial heme biosynthesis cascade is of particular interest due to its direct role in 5-ALA-induced intraoperative fluorescence. Disruption of this pathway involves inhibition of key mitochondrial enzymes—CPOX and PPOX, as shown in Figure 5A. While 2-HG directly suppresses PPOX activity, its interference with CPOX appears to be indirect, likely stemming from broader disturbances in redox balance and mitochondrial integrity [26]. This enzymatic blockade, driven by IDH mutations, impairs the production of PpIX, the fluorescent metabolite crucial for visualizing tumor margins during surgery under blue light. By contrast, IDH-wildtype gliomas retain a functional biosynthetic pathway, enabling effective PpIX accumulation and generating strong intraoperative fluorescence.

Despite their generally favorable clinical course and prognosis, the fluorescence deficit observed in IDH-mutant tumors has practical implications for neurosurgical management. Reduced PpIX synthesis leads to diminished or absent intraoperative fluorescence, which significantly complicates the surgical resection of IDH-mutant tumors, as they are often difficult to distinguish from surrounding healthy brain tissue. This fluorescence attenuation poses a challenge to achieving GTR—an outcome that remains critical for long-term disease control. This limitation highlights the need for novel strategies to improve intraoperative visualization. Given these challenges, vorasidenib—a selective inhibitor of mutant IDH1 and IDH2—may represent a promising and potentially transformative adjunct in the surgical management of low-grade gliomas.

### 4.1. Vorasidenib: A Targeted IDH Inhibitor Redefining Glioma Metabolism

Building on its metabolic mechanism of action, vorasidenib has demonstrated significant biochemical effects in both preclinical models and clinical trials, particularly through effective suppression of α-ketoglutarate to the oncometabolite 2-HG [13,25,27]. The compound binds the active sites of the mutant enzymes, inhibiting their neomorphic activity both in the cytoplasm (IDH1) and mitochondria (IDH2) [12]. A clinical study has demonstrated that vorasidenib achieves a reduction greater than 90% in 2-HG concentrations in both serum and tumor tissue, indicating effective metabolic modulation [13]. In this landmark translational study, Mellinghoff et al. [13] also investigated the effects of perioperative vorasidenib administration in patients with IDH-mutant gliomas. Patients received the drug for a period ranging from several days to several weeks before planned surgical resection. Subsequent analysis of resected tumor samples revealed significant and reassuring reductions in 2-HG levels, along with pharmacodynamic evidence of reactivation of α-ketoglutarate-dependent enzymes. Additionally, the study identified epigenetic, metabolic, and microenvironmental changes consistent with partial reversion of the tumor’s IDH-driven molecular phenotype. This was the first study to demonstrate that even short-term preoperative exposure to vorasidenib can exert measurable biological effects at the tumor level, supporting the integration of targeted metabolic therapy into surgical management strategies.

Ongoing clinical trials, with a primary focus on WHO grade 2 IDH-mutant gliomas, are actively exploring the potential of vorasidenib. Early data suggest that vorasidenib may be less effective in tumors exhibiting radiographic contrast enhancement, a radiological feature commonly associated with higher-grade transformation and a more aggressive tumor phenotype [12].

### 4.2. IDH Mutations and Vorasidenib: Understanding the Paradox of Favorable Genetics and Targeted Therapy

A clinically relevant dilemma arises—if IDH mutations in gliomas are associated with favorable prognosis and prolonged survival, how can vorasidenib—a drug that inhibits effects of these mutations—further improve therapeutic outcomes? Vorasidenib exerts its therapeutic effect by inhibiting the aberrant production and accumulation of 2-HG, which drives a cascade of deleterious molecular events, including secondary genetic alterations, impaired cellular differentiation, mitochondrial dysfunction, oxidative stress, and widespread metabolic dysregulation [23,25]. Prolonged exposure to these aberrations can lead to progressive epigenetic destabilization and ultimately facilitate malignant progression to higher-grade phenotypes [24,28]. Thus, although IDH mutations are initially prognostically favorable, their long-term metabolic consequences may promote tumor evolution. By inhibiting the activity of mutant IDH enzymes, vorasidenib enables the reactivation of α-ketoglutarate-dependent pathways, such as DNA and histone demethylation, improvement in mitochondrial homeostasis, and partial restoration of differentiation capacity in glioma cells [24,27,28]. By mitigating these pathological processes, vorasidenib may contribute to stabilizing the tumor microenvironment and potentially delaying malignant progression.

This hypothesis is supported by recent clinical data. In the phase 3 INDIGO trial, which enrolled patients with IDH-mutant grade 2 gliomas who had not undergone prior radiotherapy or chemotherapy, vorasidenib more than doubled the median PFS compared to placebo (27.7 months vs. 11.1 months), while significantly delaying the initiation of additional therapeutic interventions [13].

### 4.3. Implications for Future Research: Exploring the Potential of Vorasidenib to Enhance 5-ALA-Guided Fluorescence

To date, no preclinical or clinical studies have directly investigated the potential interaction between vorasidenib and 5-ALA-induced fluorescence. However, based on established biochemical mechanisms, it is reasonable to hypothesize that inhibition of 2-HG production by vorasidenib could restore α-ketoglutarate-dependent mitochondrial function and reactivate key enzymes involved in heme biosynthesis. In particular, enhanced activity of CPOX and PPOX may increase the accumulation of fluorescent PpIX following 5-ALA administration. This hypothetical pathway is illustrated in Figure 5B, summarizing the potential molecular mechanisms involved.

Clinically, this effect may be especially relevant in low-grade IDH-mutant gliomas, where intraoperative fluorescence is typically weak or absent, posing a challenge to achieve GTR [29,30]. Improved visualization of tumor margins intraoperatively represents a significant advancement in neurosurgical oncology, enabling more precise and safer resections, which could lead to prolonged PFS and OS. Further translational and clinical studies are warranted to evaluate whether administration of vorasidenib can augment 5-ALA-induced fluorescence and thereby improve intraoperative tumor visualization in patients with IDH-mutant gliomas. Any potential clinical implementation of this concept would require a reliable preoperative determination of IDH mutation status. This could theoretically be achieved by stereotactic biopsy in newly diagnosed cases. However, the most feasible scenario would be in recurrent IDH-mutant gliomas, where molecular status has already been established from prior surgery, as illustrated by the perioperative trial of Mellinghoff et al. [13]. If validated, this approach might enhance intraoperative visualization and establish a novel paradigm in glioma surgery by integrating metabolic therapy with fluorescence-guided techniques.

### 4.4. Limitations

This literature review has certain limitations. The included studies demonstrated substantial methodological heterogeneity, particularly in terms of fluorescence assessment protocols. Most investigations relied on subjective visual grading of intraoperative fluorescence, while only a limited number employed objective quantification methods. Furthermore, critical outcome measures—such as the extent of resection, PFS, and OS—were inconsistently reported across studies, thereby limiting the robustness and generalizability of the synthesized findings.

A further limitation concerns the exploratory hypothesis regarding vorasidenib, which remains entirely speculative and is not supported by preclinical or clinical data. Experimental validation is urgently needed, particularly in IDH-mutant low-grade glioma cell lines, to assess PpIX accumulation and fluorescence following combined exposure to 5-ALA and vorasidenib. The development of a standardized experimental protocol—including optimized dosing regimens, timing of drug administration, and fluorescence measurement intervals—will be essential to generate reproducible and clinically meaningful data. Notably, preclinical studies addressing this question are currently being conducted in our laboratory, which are expected to provide the first empirical evidence to support or refute this hypothesis.

## 5. Conclusions

This systematic review demonstrates that the effectiveness of 5-ALA-guided fluorescence in glioma surgery is strongly dependent on WHO grade and variably influenced by IDH mutation status, with markedly reduced detection in many IDH-mutant tumors.

For the first time, we propose the exploratory hypothesis that vorasidenib may enhance intraoperative fluorescence in this setting by restoring heme biosynthesis pathways. While this concept remains speculative, it represents a compelling target for future translational research and requires rigorous experimental validation.

## Figures and Tables

**Table 1:** Characteristics of the included studies.

	Bianconi et al., 2025 [14]	Specchia et al., 2021 [15]	Muther et al., 2022 [16]	Kim et al., 2021 [17]	Hickmann et al., 2015 [18]	Ohba et al., 2020 [19]	Zeppa et al., 2022 [20]
**Study methodology**	Retrospective single-center cohort study	Retrospective study	Retrospective single-center cohort study	Retrospective single-center study	Retrospective controlled cohort study	Retrospective observational study	Retrospective single-center cohort study
**Study group**	112 patients: 69 IDH-mutant astrocytomas (28 WHO G2, 41 WHO G3). 43 IDH-mutant oligodendrogliomas (24 WHO G2, 19 WHO G3)	44 GBM patients (16 IDH-mutant, 28 IDH-wildtype)	179 patients: 113 WHO G2, 66 WHO G3	Final study cohort: 25 patients GBM IDH-wildtype	5-ALA group: 58 Control group: 65 All patients with recurrent gliomas	104 patients, (68 underwent initial surgery, 36 were reoperated)	99 GBM patients (WHO G4)
**5-ALA fluorescence rate -** **WHO G4**	Not included	IDH-mutant Grade 0: 8/16 (50%) Grade 1: 7/16 (43.75%) Grade 2: 1/16 (6.25%) IDH-wildtype Grade 0: 1/28 (3.6%) Grade 1: 4/28 (14.3%) Grade 2: 23/28 (82.1%)	Not included	Pink (*n* = 14) 50%, blue (*n*=122) 28.7%	Fluorescence in 36/38 (94.7%)	Fluorescence in 57/59 (96.6%)	100%
**5-ALA fluorescence rate -** **WHO G3**	43.3% (26/60)	---	75–85%	Not included	17/25 (68.0%)	20/26 (76.9%)	---
**5-ALA fluorescence rate -** **WHO G2**	25.0% (15/60)	---	16–20%	Not included	Not reported	5/19 (26.3%)	---
**Association with IDH mutations**	No significant correlation between IDH mutation status and fluorescence (*p* = 0.94)	Grade 2 in 66.7% of IDH-wildtype, IDH-mutant: mostly Grade 0/1. Not statistically significant (*p* = 0.25)	Fluorescence did not significantly correlate with IDH mutation status (*p* = 0.814)	Study focused exclusively on IDH-wildtype GBM	100% of non-fluorescent tumors were IDH-mutant, vs. 48.9% of fluorescent tumors (*p* = 0.005)	IDH mutations were present in 35.6% of tumors (37/104), fluorescence was significantly lower in IDH-mutant tumors compared to IDH-wildtype across all WHO grades (51.4% vs. 94.0%, *p* < 0.01)	all cases IDH–wildtype
**Impact on surgical resection**	Gross total resection (GTR) achieved in 71% (80/112); fluorescence was not significantly correlated with extent of resection	GTR rate not directly assessed	GTR rate not directly assessed	No correlation between residual fluorescence and tumor infiltration	Complete resection (EOR ≥ 98%) was achieved in: 58.8% of fluorescent cases, 50% of non-fluorescent cases (not significant, *p* = 0.622)	Not reported	≥90% extent of resection: 87.5% (5-ALA), 77.3% (SF), 80% (combined); no significant difference (*p* = 0.783)
**Fluorescence technology/** **surgical equipment**	Leica M530 OHX microscope with FL400 (5-ALA) and FL560 (FS) filters	Pentero Zeiss Microscope with a proper UV 400 nm filter	Zeiss Meditech Pentero, Zeiss Pentero 900, both equipped with BLUE400 module	Zeiss BLUE400 filter system	Blue light operating microscope with BLUE400 module	Pentero surgical microscope (Carl Zeiss Meditec, Germany) with the BLUE400 module	Operating microscopes with dedicated fluorescence filters (5-ALA and SF); specific device not reported.
**Long-term outcomes**	5-ALA positivity associated with ~2.5-fold higher risk of shorter OS (*p* = 0.009) and PFS (*p* = 0.004); FS positivity with ~2.7-fold higher risk of shorter OS (*p* = 0.014), PFS not significant	Not reported	Not reported	Median PFS: 12.5 ± 2.1 months Median OS: 21.1 ± 3.5 months	PFS: 5-ALA group: 10.7 months Control: 10.6 months (not significant) OS: 5-ALA group: 17.6 months Control: 14.6 months (*p* = 0.025)	Not reported	Median OS: 20.0 months (5-ALA), 12.3 months (SF), 18.1 months (combined); overall cohort median OS 14.9 months; no significant differences
**Study limitations**	Lack of quantitative fluorescence assessment, no standardized grading of fluorescence intensity	No quantitative fluorescence assessment, lack of outcome data	Lack of outcome data	Small sample size, lack of quantitative fluorescence assessments	Subjective fluorescence assessment, no histological confirmation of residual fluorescent areas	Relatively small sample size, especially for subgroup analyses. Lack of outcome data	Limited generalizability (IDH-wildtype GBM only), small subgroup sizes

## Abbreviations

The following abbreviations are used in this manuscript:

5-ALA	5-aminolevulinic acid
IDH	isocitrate dehydrogenase
PFS	progression-free survival
OS	overall survival
GTR	gross total resection
WHO	World Health Organization
PGB	porphobilinogen
CPOX	coproporphyrinogen oxidase
PPOX	protoporphyrinogen oxidase
PpIX	protoporphyrin IX
FECH	ferrochelatase
2-HG	2-hydroxyglutarate
FDA the U.S.	Food and Drug Administration
PRISMA	Preferred Reporting Items for Systematic Reviews and Meta-Analysis
GBM	Glioblastoma
LGGs	low-grade gliomas
MGMT	O^6^-Methylguanine-DNA Methyltransferase
CE	contrast enhancement
LOOCV	leave-one-out cross-validation
EOR	extent of resection
HO-1	heme oxygenase-1
TCA	tricarboxylic acid cycle
